# Serological Screening of Influenza A Virus Antibodies in Cats and Dogs Indicates Frequent Infection with Different Subtypes

**DOI:** 10.1128/JCM.01689-20

**Published:** 2020-10-21

**Authors:** Shan Zhao, Nancy Schuurman, Malte Tieke, Berit Quist, Steven Zwinkels, Frank J. M. van Kuppeveld, Cornelis A. M. de Haan, Herman Egberink

**Affiliations:** a Virology Section, Infectious Diseases and Immunology Division, Department of Biomolecular Health Sciences, Faculty of Veterinary Medicine, Utrecht University, Utrecht, The Netherlands; University of Tennessee at Knoxville

**Keywords:** influenza A virus, cat, dog, serology, SpyCatcher, hemagglutinin, nanoparticle

## Abstract

Influenza A viruses (IAVs) infect humans and a variety of other animal species. Infections with some subtypes of IAV were also reported in domestic cats and dogs. In addition to animal health implications, close contact between companion animals and humans also poses a potential risk of zoonotic IAV infections. In this study, serum samples from different cat and dog cohorts were analyzed for IAV antibodies against seven IAV subtypes, using three distinctive IAV-specific assays differing in IAV subtype-specific discriminatory power and sensitivity. Enzyme-linked immunosorbent assays against the complete hemagglutinin (HA) ectodomain or the HA1 domain were used, as well as a novel nanoparticle-based, virus-free hemagglutination inhibition assay. Using these three assays, we found cat and dog sera from different cohorts to be positive for antibodies against one or more IAV subtypes and/or strains. Cat and dog serum samples collected after the 2009 pandemic H1N1 outbreak exhibit much higher seropositivity against H1 compared to samples from before 2009. Cat sera, furthermore, displayed higher reactivity for avian IAVs than dog sera. Our findings show the added value of using complementary serological assays, which are based on reactivity with different numbers of HA epitopes, to study IAV antibody responses and for improved serosurveillance of IAV infections. We conclude that infection of cats and dogs with both human and avian IAVs of different subtypes is prevalent. These observations highlight the role of cats and dogs in IAV ecology and indicate the potential of these companion animals to give rise to novel (reassorted) viruses with increased zoonotic potential.

## Abstract (precis)

Influenza A viruses (IAVs) infect humans and a variety of other animal species. Infections with some subtypes of IAV were also reported in domestic cats and dogs. In addition to animal health implications, close contact between companion animals and humans also poses a potential risk of zoonotic IAV infections. In this study, serum samples from different cat and dog cohorts were analyzed for IAV antibodies against seven IAV subtypes, using three distinctive IAV-specific assays differing in IAV subtype-specific discriminatory power and sensitivity.

## INTRODUCTION

Influenza A viruses (IAVs) are enveloped, negative-sense segmented RNA viruses that belong to the *Orthomyxoviridae* family. They cause seasonal epidemics, pandemics, and sporadic zoonotic infections (1). Wild aquatic birds are the natural host reservoir, but IAVs have been isolated from not only humans but also from many other species, including cats, dogs, horses, pigs, mink, ferrets, foxes, marine mammals, and domestic birds (2). Thus, IAVs can easily cross the species barrier to infect new species. IAVs contain two glycoproteins expressed on the viral membrane, the hemagglutinin (HA) and the neuraminidase (NA). So far, 18 HA and 11 NA subtypes have been identified from IAVs circulating in birds and mammals, which through reassortment give rise to many viral subtypes with different HA/NA combinations (3–6). In addition, IAVs often exhibit within-host genetic diversity because of their high mutation rate, efficient replication, and large virus population sizes (7). Thus far, IAV surveillance is mostly limited to birds, humans, and swine. However, in view of the large number of mammalian species in which IAVs have been found and the large zoonotic potential of IAVs, surveillance should not be restricted to these few species.

Domestic cats and dogs are increasingly being recognized as hosts of IAVs. Previous studies have confirmed isolation of different avian and human IAVs of different subtypes in cats, including human-derived pandemic H1N1 (H1N1pdm09), which emerged in 2009 (8), and avian-derived H7N2, which caused an outbreak in a shelter in the United States in 2016 and 2017 (9). The latter led to the first confirmed case of IAV transmission from cat to human (10). Dogs are also susceptible to different IAVs. IAV outbreaks in dogs have been reported for equine-derived H3N8 viruses in 2004 (11), as well as for avian-derived H3N2 viruses (12, 13). Occasional spillover events of other IAV subtypes (H1N1pdm09, avian H5N1, and H5N2) to dogs have also been reported (14). Notably, the majority of IAV infections in cats and dogs were found in Asia and North America, while in Europe only infections with H1N1pdm09, H3N8, and H5N1 have been reported (15–19). These observations stress the potential role of cats and dogs in IAV circulation and the importance of IAV surveillance in these domestic animals.

Being the major surface glycoprotein of influenza virus, HA mediates binding of the virus to cell surface receptors and virus-cell fusion. It is also the prime target for neutralizing antibodies (20–22). HA is a homotrimer, with each protomer comprised by two functional interdependent subunits, HA1 and HA2. Of note, the immunodominant globular head domain of HA formed by the central part of HA1, which contains the receptor-binding site (RBS), is the most variable IAV antigen. The more conserved and immune-subdominant stalk domain of HA is formed by the HA2 subunit, along with N- and C-terminal regions of the HA1 (20, 22). Antibodies to the head domain of HA can be strongly neutralizing but are mostly subtype or even strain specific, whereas antibodies against the stalk domain often show cross-reactivity within and across HA subtypes (23).

Serological screening can be used to support clinical diagnosis of IAV infection, herd immunity profiling, and monitoring of vaccine compliance. Moreover, it poses a foundation for seroprevalence-based epidemiological studies. Commonly used serological methods include hemagglutination inhibition (HI) assays, which measure antibody titers by inhibition of the agglutination of erythrocytes, as well as enzyme-linked immunosorbent assay (ELISA) and virus neutralization (VN) assay that assess the presence of neutralizing antibodies. Incongruences between these different IAV serological assays have been reported (24, 25). VN and HI assays are generally recognized as “gold standards” but require culturing of potentially dangerous viruses. In addition, conventional HI, while being highly subtype specific, is relatively insensitive when detecting antibodies in animal and human sera (26, 27).

In the present study, we developed a pipeline of serological assays which allow broad or specific analysis of IAV-specific antibody responses. In this pipeline, serum samples are tested first with HA- and HA1-specific ELISAs and subsequently analyzed by nanoparticle-based, virus-free HI assays. Our study shows the seroprevalence of antibodies to IAVs of both avian and human origin in European cats and dogs, which underscores the potential role of these domestic animals as IAV “mixing vessels.”

## MATERIALS AND METHODS

### Serum samples.

Sheep polyclonal reference antisera (09/142 and 03/212) against purified HA of A/California/7/09 (anti-H1_2009_) and A/Wyoming/03/03 virus (anti-H3_N2_) were provided by the National Institute for Biological Standards and Control (London, UK). Goat polyclonal reference antiserum (NR-34586) to the HA of A/duck/Shantou/1283/2001 (anti-H3_N8_) and rabbit polyclonal reference antiserum (NR-48765) to the HA of A/Shanghai/1/2013 (anti-H7_N9_) were provided by Biodefense and Emerging Infectious Research Resources Repository (BEI Resources, Washington, DC). Chicken polyclonal antiserum against H5N8 A/Chicken/Netherlands/SP00213/2017 (anti-H5_N8_) or H9N2 A/Chicken/Saudi Arabia/SP02525/3AAV/2000 (anti-H9_N2_) was provided by GD Animal health Deventer, the Netherlands. Seronegative normal goat serum (Invitrogen, Carlsbad, CA), normal rabbit serum (Invitrogen) and mock-vaccinated chicken serum from previous study (28) were included as negative controls.

For the serological screening, a total of 321 feline samples and 222 canine samples were included. Feline samples were obtained from three different cohorts: samples send to the University Veterinary Diagnostic Laboratory of Utrecht University before 2009 (serum samples, *n* = 68) and in 2019 (plasma samples, *n* = 131), and samples from abandoned or stray cats at shelters across the Netherlands of 2016 (serum samples, *n* = 122). Canine samples were collected from two cohorts: samples collected before 2009 (serum samples, *n* = 68) and between May and July 2019 (serum samples, *n* = 154). All samples were collected for diagnostic purposes independent to this study. In addition, sera of specific-pathogen-free (SPF) cats and dogs shown also to be negative for IAV were included as negative controls. All serum samples were stored at –20°C until tested.

### Cells and viruses.

Human embryonic kidney 293 cells stably expressing the SV40 large T antigen (HEK-293T), *N*-acetylglucosamine transferase I-deficient HEK293S GnTI^–^ cells (29) and type II Madin-Darby canine kidney (MDCK-II) cells were maintained in Dulbecco modified Eagle medium (Lonza, Basel, Switzerland) supplemented with 10% fetal bovine serum (Bodinco, Alkmaar, The Netherlands), penicillin (100 IU/ml), and streptomycin (100 μg/ml).

Influenza virus A/Netherlands/602/2009 (H1N1) (30) was propagated in MDCK-II cells as described previously (31) and stored at −80°C until use. Prior to the use in hemagglutination/hemagglutination inhibition assays, virus was inactivated via UV radiation using UV Stratalinker 1800 (Stratagene) at 50,000 μJ.

### Constructs design, protein expression, and purification.

**FIG 1:**
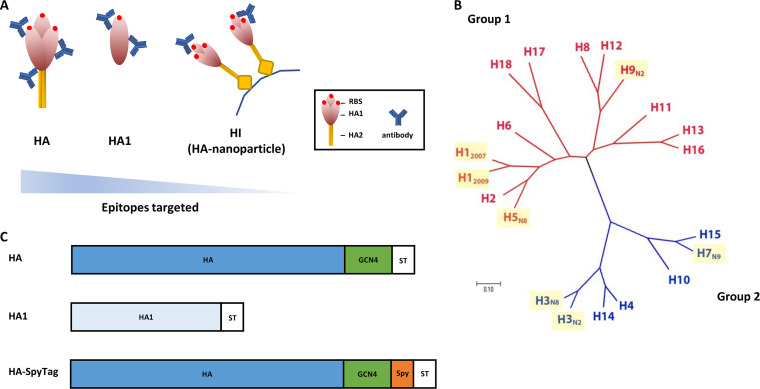
Recombinant soluble HA proteins used in this study. (A) Schematic depiction of antibody interaction with HA trimeric protein, HA1 protein, and HI (HA-conjugated nanoparticle). The triangle indicates the different number of epitopes targeted in the different assays. (B) Phylogenetic tree indicating genetic relationship of HAs from different influenza A subtypes. HA proteins used in this study are marked in yellow. (C) Schematic representation of the recombinant HA proteins, HA1 proteins, and HA-SpyTag proteins. The HA protein contains the HA1 and HA2 subunits fused to the GCN4 isoleucine zipper trimerization motif (GCN4) and Strep-tag II (ST). The HA1 protein contains the HA1 subunit of HA protein and ST. The HA-SpyTag protein has a schematic structure similar to that of the HA protein but carries an additional peptide SpyTag (Spy).

Expression constructs of recombinant HA proteins are shown schematically in Fig. 1. Human codon-optimized HA ectodomain (amino acids 24 to 530; H1 numbering) encoding cDNAs (GenScript) of A/California/04/2009 (H1N1) (GenBank accession no. ACS45035.1, referred to as H1_2009_), A/Kentucky/UR06-0258/2007 (H1N1) (GenBank accession no. ABX58635.1, referred to as H1_2007_), A/Fujian/411/2002 (H3N2) (GenBank accession no. AFD64223.1, referred to as H3_N2_), A/canine/Florida/242/2003 (H3N8) (GenBank accession no. ABA39842.1, referred to as H3_N8_), A/Eurasian wigeon/Netherlands/1/2014 (H5N8) (GenBank accession no. AKH60771.1, referred to as H5_N8_), A/Anhui/1/2013 (H7N9) (GISAID isolate EPI439509, referred to as H7_N9_), and A/turkey/England/13437/2013 (H9N2) (GenBank accession no. ALR82074.1, referred to as H9_N2_) were cloned into a pFRT/pCD5 expression plasmid as described previously (32, 33). Briefly, the HA gene was cloned in an expression vector in frame with a sequence encoding a CD5 signal peptide, a GCN4-isoleucine-zipper trimerization (GCN4) (34) and a Strep-tag II (ST) for purification (WSHPQFEK; IBA GmbH, Göttingen, Germany). In order to produce HA ectodomain trimers (HA-SpyTag) to be conjugated to its protein partner SpyCatcher, the constructs of HA were designed as described above, with the addition of a sequence encoding a short (13-residue) SpyTag between GCN4 domain and ST encoding sequences (35). To generate the head domain HA1 monomer (amino acids 24 to 320; H1 numbering), the sequence encoding the corresponding HA1 domain was cloned into pFRT expression vector flanked by the sequence encoding CD5 N-terminal signal peptide and ST.

HA and HA1 expression plasmids conjugated to polyethyleneimine (Polysciences, Inc., Warrington, PA) were transfected into HEK293T cells to produce soluble proteins for ELISAs. HA-SpyTag-encoding plasmids were expressed in HEK293S GnTI^–^ cells instead of HEK293T cells, to increase the affinity of HA for its receptor (36). At 6 to 7 days posttransfection, cell supernatants containing soluble HA and HA1 proteins were harvested, and proteins were purified using Strep-Tactin Sepharose beads according to the manufacturer’s instructions (IBA). Purified proteins were quantified by NanoDrop spectrophotometry (Thermo Fisher Scientific, Inc., Waltham, MA), analyzed on an SDS-PAGE gel under reducing conditions, and visualized with GelCodeBlue stain reagent (Thermo Fisher Scientific). Purified proteins were stored at −80°C until further usage.

### Expression and purification of SpyCatcher-mi3 nanoparticles.

pET28a-SpyCatcher-mi3 is kindly provided by Mark Howarth (Addgene plasmid, catalog no. 112255; RRID: Addgene_112255). The pET28a expression plasmid was transformed into *Escherichia coli* BL21 (Agilent) similarly as described previously (37). After 16 h of incubation at 37°C, a single colony was picked into a starter culture in Luria-Bertani (LB) medium from an LB agar plate containing 50 μg/ml kanamycin, followed by incubation overnight at 37°C with shaking at 220 rpm. Next, the entire 10-ml starter culture LB medium was diluted into 1 liter of LB culture medium, followed by incubation at 37°C with shaking at 200 rpm. The cultures were induced with 0.5 mM IPTG (isopropyl-β-d-thiogalactopyranoside) until the *A*_600_ reached 0.8 and were then grown for another 16 to 20 h at 22°C with shaking at 200 rpm. The pellet derived from 500 ml of culture was resuspended in 10 ml of lysis buffer (25 mM Tris-HCl [pH 8.5], 150 mM NaCl, 0.1 mg/ml lysozyme, 1 mg/ml complete mini-EDTA-free protease inhibitor [Sigma-Aldrich], and 1 mM phenylmethanesulfonyl fluoride [Sigma-Aldrich]) at 4°C and rotated at 25°C for 1 h. After sonication on ice, the lysis mixture was spun down at 14,000 × *g* for 30 min at 4°C. For SpyCatcher-mi3 nanoparticle (NP) purification, Capture Select C-tag Affinity Matrix (Thermo Fisher Scientific) was added, followed by incubation for 1 h at 4°C on a tube roller. NPs were then eluted with elution buffer (20 mM Tris-HCl [pH 7.4] with 2 M MgCl_2_ at 4°C) according to the manufacturer’s instructions. All eluted NPs were pooled and concentrated by using a 100-kDa MWCO Vivaspin ultrafiltration unit (Sartorius). NPs were then separated into aliquots and stored at −80°C until further usage.

### Western blot analysis.

To assess the antigenicity of the expressed HA and HA1 proteins, 1 μg of purified HA or HA1 was loaded on an SDS-PAGE gel and subsequently electroblotted onto a polyvinylidene difluoride membrane. Membranes loaded with all HA or HA1 proteins were then incubated separately with reference antisera at a 1:200 dilution at room temperature for 1 h. The antigen-antibody interaction was detected with horseradish peroxidase (HRP)-conjugated secondary antibody (1:4,000 for rabbit anti-goat/sheep immunoglobulins/HRP and swine anti-rabbit immunoglobulins/HRP from Dako Agilent [Santa Clara, CA]; 1:4,000 for goat anti-chicken immunoglobulin G/HRP from Southern Biotech Associates, Inc. [Birmingham, AL]) and visualized by using Pierce ECL Western blotting substrate (Thermo Fisher) according to the user manual.

### Enzyme-linked immunosorbent assay.

High-binding microtiter plates (Greiner Bio-One BV, Alphen aan den Rijn, The Netherlands) were coated overnight at 4°C with 1 μg/ml HA protein or 2 μg/ml HA1 protein (100 μl per well, diluted in phosphate-buffered saline [PBS; pH 7.4]). After three washes with washing buffer (PBS containing 0.05% Tween 20), the plates were blocked for 2 h at 37°C with blocking buffer (PBS containing 5% milk powder [Protifar; Nutricia, Zoetermeer, The Netherlands] and 0.05% Tween 20). Protein coating efficiency was assessed based on the binding of StrepMAB-Classic (Strep-tag II specific monoclonal antibody) conjugated to HRP (IBA) in a direct ELISA. To determine the half-maximal effective concentration (EC_50_) of each reference antiserum against its homologous HA and HA1 proteins, 2-fold serial dilutions of serum samples were tested in an indirect ELISA format. Serum samples were diluted in blocking buffer (starting from 1:50) and incubated in HA/HA1 coated plates at 37°C for 1 h. After washing, the plates were further incubated with diluted HRP-conjugated secondary antibody using the same concentration as that used for the Western blot analysis described above at 37°C for 1 h. The peroxidase reaction was then visualized by adding TMB Super Slow One-Component HRP Microwell substrate (BioFX; Surmodics IVD, Inc., Eden Prairie, MN) for 10 min. The reaction was then quenched with 12.5% sulfuric acid, and the optical densities (OD) were measured at 450 nm. The OD values were plotted against dilution ratios, and the EC_50_ of each reference antiserum against the homologous HA/HA1 was calculated by using a four-parameter logistic regression curve-fitting method (GraphPad Prism, v7.04) and expressed as dilution ratios. Experiments were performed in triplicate.

For the screening of serum samples, indirect ELISAs with different HA or HA1 proteins as antigens were conducted. In brief, reference antisera at EC_50_ or cat and dog sera at a 1:200 dilution were incubated for 1 h in HA/HA1-coated plates; after washing, the plates were further incubated with HRP-conjugated secondary antibodies (reference antisera were used as described above: at 1:4,000 for goat anti-cat IgG/HRP [Rockland Immunochemicals, Inc., Pottstown, PA] and at 1:6,000 for goat anti-dog IgG/HRP [Cappel, Costa Mesa, CA]), followed by TMB development and sulfuric acid quenching. Seronegative goat, rabbit, and chicken sera and sera from SPF cats and dogs were taken as negative controls to determine the cutoff values. Sera with OD values >5-fold the OD value of the corresponding negative sera were considered positive. ELISA results are expressed as ratios of the OD_450_ value of the analyzed sera and the positive cutoff values (OD_450_ value of the serum/positive cutoff value) (38, 39). All seven HA or seven HA1 proteins were coated on the same ELISA plates, making it easy to screen and compare the OD values of individual sera in one assay.

Part of the serum samples was also analyzed with a commercial competitive nucleoprotein ELISA (ID screen influenza A antibody competition; IDvet, Grabels, France) according to the manufacturer’s user manual.

### Hemagglutination and hemagglutination inhibition assays with HA-conjugated SpyCatcher-mi3 nanoparticles.

Purified SpyCatcher-mi3 nanoparticles were incubated with a 1.5-fold molar excess of HA-SpyTag (a single HA-SpyTag for conjugating homotypic nanoparticles) for 36 h at 25°C in reaction buffer (25 mM Tris-HCl [pH 8.5], 150 mM NaCl [pH 8.5]). The coupling efficiency was analyzed by reducing an SDS-PAGE gel stained with GelCode Blue (Thermo Fisher Scientific). The HA-conjugated SpyCatcher-mi3 nanoparticles (HA-NPs) were then 2-fold serially diluted and mixed 1:1 with human erythrocytes (0.5% in PBS). Hemagglutination was assessed after 2 h of incubation on ice, and hemagglutinating units (HAU) were calculated for each HA-NP.

To study the HI ability of serum samples, HI assays were carried out according to the World Health Organization *Manual on Animal Influenza Diagnosis and Surveillance* (40). Before analysis, samples were first treated with receptor-destroying enzyme (RDE) prior to reaction with HA-NPs. Serum samples were mixed with RDE (Denka Seiken Co., Ltd., Tokyo, Japan) in a 1:3 ratio. The serum-RDE mixture was incubated at 37°C for 16 h, followed by RDE inactivation at 56°C for 30 min. Treated sera were then 2-fold serially diluted and mixed 1:1 with PBS containing 4 HAU of HA-NPs. The mixtures were incubated at room temperature for 1 h and then mixed 1:1 with human erythrocytes (0.5% in PBS). Hemagglutination inhibition was recorded after 2 h of incubation on ice. The HI titer of a serum sample is expressed as the reciprocal of the highest serum dilution still showing inhibition of hemagglutination. Reference antisera, cat and dog sera, and negative-control sera were tested in the same fashion as described above. Serum samples with an HI titer of ≥1:20 were considered to be seropositive.

### Statistical analyses.

All statistical analyses were performed using GraphPad Prism v7.04 for Windows (GraphPad Software, La Jolla, CA). The 95% confidence interval (95% CI) was computed by the modified Wald method. Cohen’s kappa values were determined as a measure of the overall agreement. A chi-square test was used to assess the difference in the prevalence between two groups, and a *P* value of <0.05 was considered statistically significant.

## RESULTS

### Conceptual design of ELISA and HI assays for serological screening.

We devised a combination of assays, including HA- and HA1-specific ELISAs and hemagglutination inhibition (HI), which differ in the number and nature of epitopes that are targeted. As a result, they differ in the breadth of antibody detection, theoretically ranging from broad to specific for different IAV subtypes and strains (Fig. 1A). Using trimeric HA protein in ELISA allows detection of most epitopes present in both the variable HA1 and conserved HA2 subunit. Switching from HA to HA1, the range of detection will be narrowed down to HA1-specific epitopes. The HI assay only focuses on epitopes surrounding the RBS, antibody binding to which will interfere with HA-receptor interactions (Fig. 1A).

To allow evaluation of antibody responses against relevant IAV strains, we included HAs of representative IAV strains in our screens (Fig. 1B). Our library contains HAs of IAV strains that have been found in humans, cats, and/or dogs, such as H1 (human H1N1 circulating before and after 2009 (H1_2007_ and H1_2009_, respectively) and H3 (human H3_N2_ and canine H3_N8_), as well as HAs of important avian IAV subtypes (H5_N8_, H7_N9_, and H9_N2_). Collectively, these different HAs cover a large genetic and thus antigenic space (Fig. 1B).

### Production and functional assessment of HA proteins and HA-conjugated nanoparticles.

Recombinant soluble HAs were genetically fused to a Strep-tagged GCN4 domain to ensure expression in their native trimeric form (36), while HA1s were only provided with a Strep-tag (Fig. 1C). Protein expression and purification was analyzed by Western blot analysis with reference antisera of anti-H1_2009_, -H3_N2_, -H3_N8_, -H5_N8_, -H7_N9_, and H9_N2_. All HA and HA1 proteins displayed electrophoretic mobilities corresponding to their expected molecular weights and displayed reactivity with the corresponding reference sera (see Fig. S1 in the supplemental material). As expected, for some sera a cross-reactivity with other HAs could also be observed, which was particularly apparent for Anti-H3_N8_ with H3_N2_.

**FIG 2:**
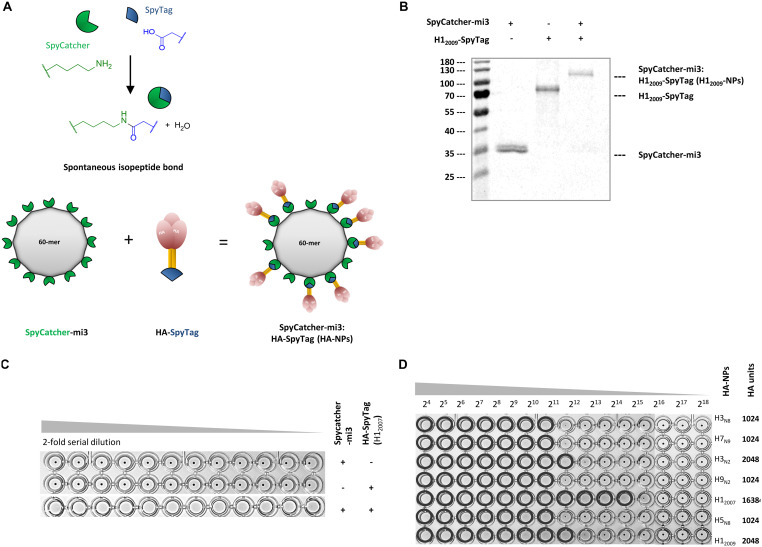
Nanoparticles displaying HAs have hemagglutination activity. (A) Cartoon representation of self-assembly of HA-nanoparticles (HA-NPs) by forming spontaneous isopeptide bonds between SpyTag-HA protein and SpyCatcher-mi3 nanoparticles. (B) SpyCatcher-mi3 NPs were incubated with HA-SpyTag at a 1:1.5 molar ratio at 25°C for 36 h, followed by analysis of HA-NP formation via reducing SDS-PAGE. (C) Hemagglutination by HA is dependent on its multivalent presentation on nanoparticles, as shown by a hemagglutination assay with human erythrocytes. (D) HA units obtained with the HA-NPs displaying different HAs.

In the present study, we also explored the possibility of performing HI assays without the need for virus propagation. Since the receptor-binding avidity of soluble trimeric HA is not high enough to efficiently agglutinate erythrocytes, multivalent presentation of HA trimers is needed (36). To achieve this, we expressed our HA proteins with a C-terminal SpyTag (Fig. 1C) and conjugated the trimeric HAs to a SpyCatcher protein genetically fused to self-assembling mi3 NPs (Fig. 2A). SpyCatcher-mi3:HA-SpyTag conjugation results in spontaneous formation of isopeptide bonds (Fig. 2A; see also reference 37). High conjugation efficiency between SpyTagged HA and SpyCatcher-mi3 was confirmed by the altered electrophoretic mobility of the conjugated proteins in polyacrylamide gels (Fig. 2B).

Next, the ability of the SpyCatcher-mi3:HA-SpyTag nanoparticles (HA-NPs) to support hemagglutination was analyzed. Strong agglutination of human erythrocytes was indeed observed for NPs displaying HA (HA-NPs) but not for trimeric HAs or “empty” NPs (Fig. 2C). H1_2007_-NPs exhibited more than 1,024 hemagglutination units at a 1 μM concentration. We next tested NPs decorated with each of the seven SpyTagged HAs, resulting in hemagglutination units between 1,024 and 13,684 (Fig. 2D). The data show that NPs decorated with trimeric HAs display high hemagglutination activity and indicate that these NPs can be used for serology screening using the HI assay.

### Validation of HA ELISA, HA1 ELISA, and NP-based HI assay with reference antisera.

**FIG 3:**
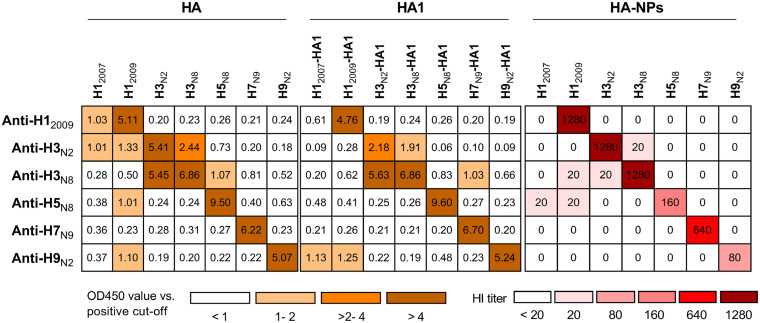
Reactivity of influenza HA type-specific antisera against different HAs. Reactivity was measured by HA or HA1 ELISA and by HI using HA-NPs. ELISA results are expressed as ratios of the OD_450_ value divided by the positive cutoff (five times the background). HI titers of <20 are represented as “0” in the figure.

The half-maximal effective concentration (EC_50_; expressed as a dilution ratio) of each reference antiserum against the matching HA or HA1 was determined in an indirect ELISA format (see Table S1 in the supplemental material). Next, all reference sera were analyzed by ELISA against all seven HA and HA1 proteins at their EC_50_. As indicated in Fig. 3, all reference antisera exhibit potent antibody response against matching HA and HA1 proteins (ratios of OD_450_ value and positive cutoff values between 2.18 and 9.60). Subtype-specific reactivity could readily be distinguished via this analysis, since the reactivity of each antiserum against HA or HA1 proteins of the corresponding subtype is at least 4-fold higher than that against the others (Fig. 3). The reactivity of the anti-H1 serum was lineage specific, since little reactivity against H1_2007_ was observed. In contrast, the two H3-specific sera reacted with both H3 proteins. Generally, in comparison to HA, much less cross-reactivity of the different sera was observed with the HA1 proteins. These data validate the recombinant HA and HA1 proteins as versatile tools for performing high-throughput ELISA screening of serum samples.

We also explored the inhibitory properties of the reference antisera with HI assays by using the different HA-NPs. HI titers obtained with different combinations of reference antisera and HA-NPs are summarized in Fig. 3. All reference antisera display prominent HI titers (80 to 1,280) against their homologous HA-NPs. Noticeably, antibody responses detected by HI is not just subtype specific but also strain specific. Thus, the two H3-specific sera only yielded high HI titers with the homologous H3-NPs (Fig. 3). We conclude that the HA- and HA1-ELISAs combined with the NP-based HI assay are complementary in the detection of humoral responses against closely and distantly related IAVs.

### IAVs seroprevalence in cats.

**FIG 4:**
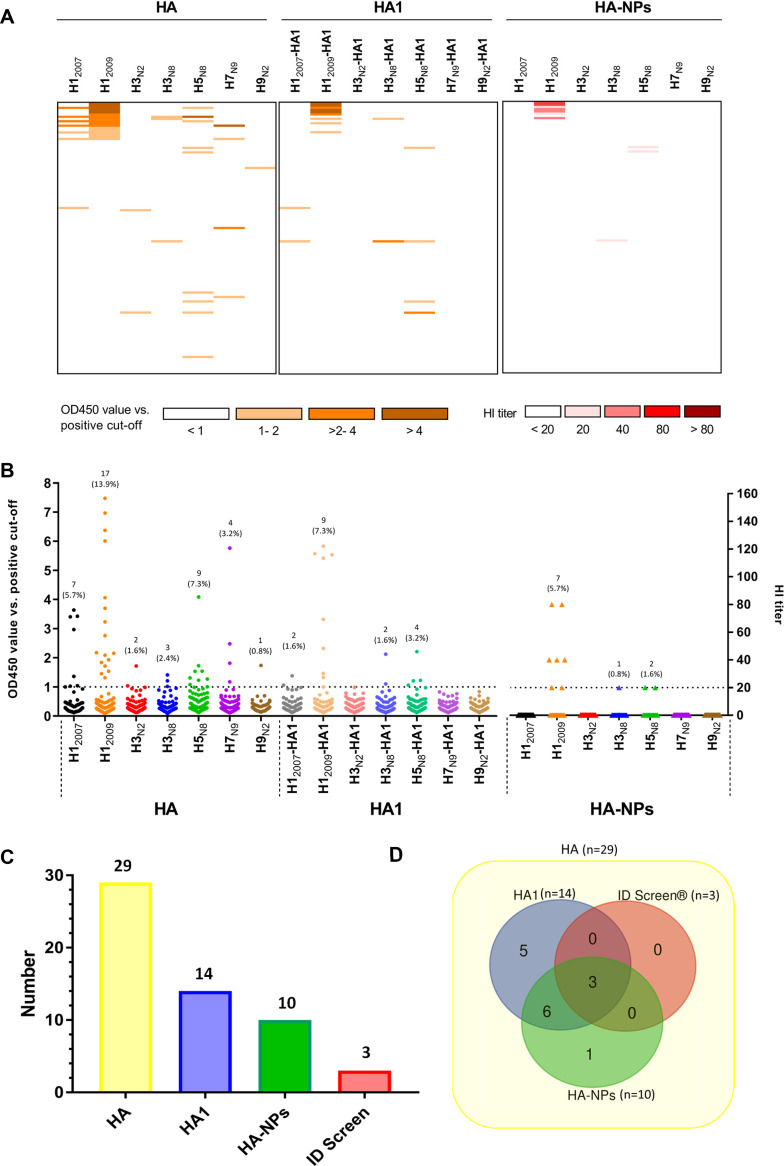
Seroprevalence of antibodies in shelter cats from 2016. Reactivity of shelter cat samples (2016 cohort, *n* = 122) against different HA proteins as measured by ELISA (the two left panels) and nanoparticle-based HI assay (right panel). Reactivity profiles of all serum samples are displayed as a heatmap (A) and a distribution dot plot (B). The number and percentage of seropositive samples are indicated for each protein, and the dashed line indicates the positive threshold. Experiments were performed as in Fig. 3. (C and D) The number of positive samples is graphed for each assay performed (C), while their overlap is shown in a Venn diagram (D).

Feline sera (*n* = 122) collected from shelters in 2016 (2016 cohort) were successively screened for influenza virus-specific antibodies using the HA and HA1 ELISAs, as well as the NP-based HI assay (Fig. 4; see also Table S2 in the supplemental material). In total, 29 of the 122 sera (23.6%) contained anti-HA antibodies detectable in the HA ELISA. The majority of responses were against H1_2009_ (17/122, 13.9%), followed by H5_N8_ (9/122, 7.3%), H1_2007_ (7/122, 5.7%), H7_N9_ (4/122, 3.2%), H3_N8_ (3/122, 2.4%), H3_N2_ (2/122, 1.6%), and H9_N2_ (1/122, 0.8%) (Fig. 4A and B). Although 21 sera reacted with only one HA protein, 8 sera exhibited reactivity against more than one HA, as shown by HA ELISA (Fig. 4; see also Table S2, rows C to E, G to I, and M). Of the eight serum samples that reacted with multiple HAs, one serum sample was positive with four HAs (Table S2, row G), four sera with three (rows D, H, and I) and three (rows C, E, and M) serum samples with two HAs. Most of the cross-reactivity occurred with sera that display reactivity with both H1_2007_ and H1_2009_ (see Table S2, rows A to J), with six of seven sera positive for H1_2007_ also being positive for H1_2009_.

Antibody responses obtained with the HA1 ELISA are expected to display less cross-reactivity. Of the 29 HA-positive sera, only 14 serum samples were positive in the HA1 ELISA (Fig. 4C; see Table S2). The pattern of positive response against HA1 is similar to HA. The majority of the sera reacted against HA1 of H1_2009_ (9/122, 7.3%), followed by H5_N8_ (4/122, 3.2%), H3_N8_ (2/122, 1.6%), and H1_2007_ (2/122, 1.6%). All HA1-positive sera are also positive for HA (see Table S2). Notably, the serum samples positive for both H1 proteins were only positive for H1_2009_-HA1, in agreement with less cross-reactivity being observed in the HA1 versus the HA ELISA. The serum sample positive for H1_2007_ only also remained positive in the H1_2007_-HA1 ELISA, although it was negative in the HA-NP HI assay (see Table S2, row K).

Subsequently, all 122 cat sera were tested in NP-based HI assays. In total, 10 serum samples were able to inhibit hemagglutination, of which seven sera were positive for H1_2009_-NPs, two for H5_N8_-NPs and one for H3_N8_-NPs (Fig. 4; Table S2). Of note, all the responses observed via HI were strictly strain specific; no cross-reactivity was observed, even with the serum samples that were positive against two or more HAs, as tested by ELISA. All but one sample positive in the HA-NP HI were also positive in the HA and HA1 ELISA. To further validate our observations, the seven H1_2009_-NP-positive sera were analyzed side by side via HI assays with HA-NPs and H1N1 virus particles, together with five cat serum samples that tested negative in this study and seven SPF cat serum samples. The data show that the HI results obtained with NPs and virus particles are nearly identical (see Table S3). In short, the results indicated that shelter cats (2016 cohort) often contain antibodies against human and/or avian influenza viruses, particularly against H1N1pdm09. The highest number of seropositivity was observed with the HA ELISA, while the lowest number was obtained with the more specific HI assay.

All the HA ELISA-positive cat sera from the 2016 cohort were also analyzed with a commercial competitive nucleoprotein ELISA kit (ID Screen ELISA) that was used in several cat and dog IAV serological studies (15, 41). Only 3 of the 29 HA-positive cat sera were determined to be positive in the ID Screen ELISA (Fig. 4; see also Table S2), and a poor agreement between HA ELISA and ID Screen ELISA was found by Kappa statistics (kappa = 0.019). A selection of 31 cat sera that were negative in our ELISA and HI were all negative in the ID screen ELISA (see Table S2, row U).

**FIG 5:**
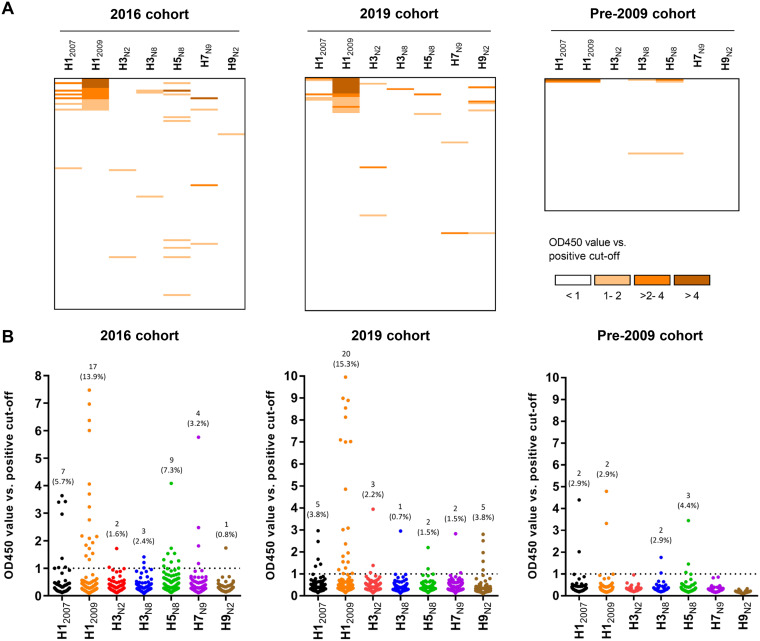
Seroprevalence of antibodies in cats from different cohorts. A comparison of reactivity against different HAs of samples from three cat cohorts—the 2016 cohort (*n* = 122; left panel), the 2019 cohort (*n* = 131; middle panel), and the pre-2009 cohort (*n* = 68; right panel)—is shown, as measured via HA ELISA. The results are presented as in Fig. 4.

Since high IAV seroprevalence was observed in samples collected from shelter cats in 2016, we also investigated antibody responses from household owned cats. Samples were collected in 2019 or pre-2009, i.e., before the H1N1pdm09 outbreak. Comparison of ELISA reactivity against HA proteins of three cat cohorts is summarized in Fig. 5. Household owned-cat samples from 2019 showed a pattern of IAV reactivity similar to that observed for shelter cats. Thus, reactivity could be observed with all HAs, with the majority of responses being against HA of H1_2009_ (15.3%). As expected, the pre-2009 cohort showed a significantly lower (*P* = 0.0141) number of positive sera for H1_2009_ compared to the post-2009 samples (Table 1). Of the three serum samples from the pre-2009 cohort that were positive for HA, two were also positive in HA1 ELISA, while none of them displayed hemagglutination inhibition (see Fig. S3 in the supplemental material). When combining all cat sera results obtained in the HA ELISAs, significantly (*P* < 0.0001) more sera were positive for H1_2009_ than for H3_N2_, both of which are HAs derived from human viruses (Table 1).

**TABLE 1:** Summary of antibody responses against different HAs by HA ELISA in cats and dogs

Species	Cohort	HA	No. of samples		Prevalence^*a*^	95% CI^*b*^
			Total	Positive		
Human IAVs						
Cat	Pre-2009	H1_2009_	68	2	0.029	0.002–0.107
	2016 and 2019	H1_2009_	253	37	0.146	0.108–0.195
	Pre-2009, 2016, and 2019	H3_N2_	321	5	0.016	0.006–0.037
Dog	Pre-2009	H1_2009_	68	0	0.000	0.000–0.064
	2019	H1_2009_	154	19	0.123	0.080–0.185
	Pre-2009 and 2019	H3_N2_	222	0	0.000	0.000–0.021
						
Avian IAVs						
Cat	Pre-2009, 2016, and 2019	H5_N8_, H7_N9_, and H9_N2_	321	25	0.078	0.053–0.113
Dog	Pre-2009 and 2019	H5_N8_, H7_N9_, and H9_N2_	222	5	0.023	0.008–0.053

a Prevalence = positive number/total sample number.

b 95% CI, 95% confidence interval (computed by the modified Wald method).

### IAV seroprevalence in dogs.

**FIG 6:**
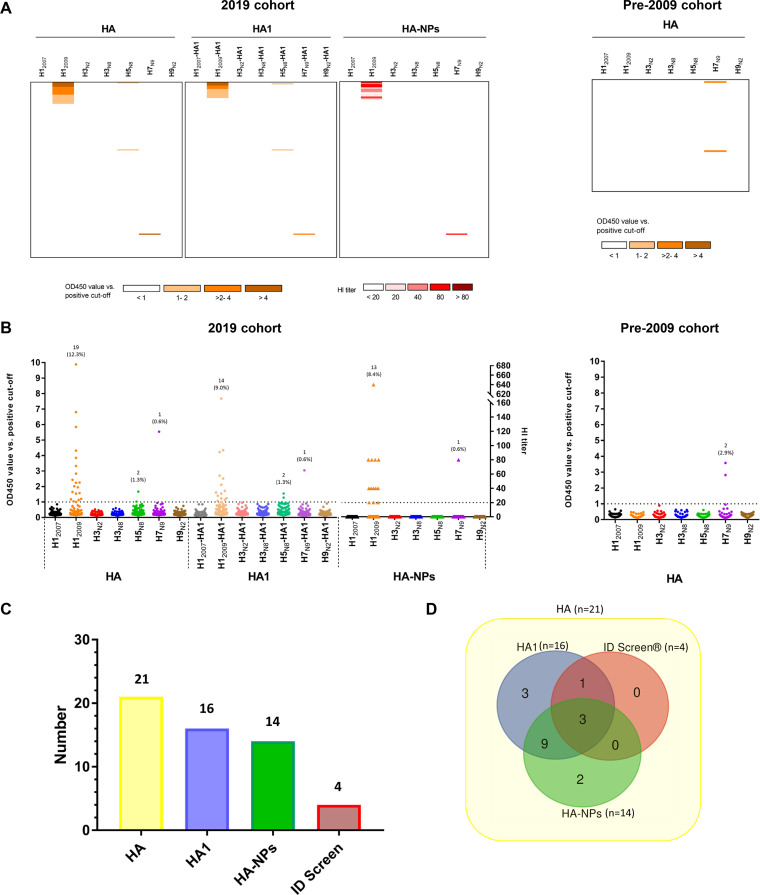
Reactivity of dog serum samples against different HA proteins. Reactivity profiles of all samples are displayed as a heatmap (A) and as a distribution dot plot (B), similarly to that shown in Fig. 4. Results for the 2019 cohort (*n* = 154) for all three assays are shown on the left. The results for the pre-2009 cohort (*n* = 68) for the HA ELISA are shown on the right. (C and D) The number of positive samples from the 2019 cohort is graphed for each assay performed (C), while their overlap is shown in a Venn diagram (D).

Canine sera (*n* = 154) collected in 2019 (2019 cohort) and prior to 2009 (pre-2009 cohort) were also screened by HA- and HA1-ELISA and NP-based HI. As summarized in Fig. 6 and Table S4, 21 serum samples (13.6%) of the 2019 cohort were positive for HA, while 16 (10.3%) and 14 (9.0%) sera were positive in HA1 ELISA and HI assay, respectively. Of the HA ELISA-positive sera, the majority were directed against H1_2009_ (*n* = 19), two serum samples were against H5_N8_, and one serum sample was positive for H7_N9_. Only one serum sample reacted with more than one HA in the HA ELISA (Fig. 6; see Table S4, row A). HA-ELISA-positive dog sera from 2019 cohort were also analyzed with ID Screen N protein ELISA (Fig. 6; see Table S4). Four of 21 dog sera tested positive, and a poor agreement between HA ELISA and ID Screen ELISA was found for dog sera (kappa = 0.289) similar to the results obtained with the cat sera. Fourteen ELISA- and HI-negative dog sera all tested negative in the ID screen ELISA (see Table S4, row K). The pre-2009 significantly differed (*P* = 0.0038) from the 2019 cohort, since no reactivity against H1_2009_ was found (Fig. 6 and Table 1). Similar to the 2019 cohort, the pre-2009 cohort contained two samples that were positive for H7 in all assays (see Fig. S3).

Comparison of the results obtained with the cat and dog sera (Table 1) indicates that sera from these two species did not differ in the seroprevalence of H1 antibodies, which for both species resulted in a significantly (*P* < 0.0001 in both cases) higher number of positive sera for H1_2009_ than for H3_N2_. Several cat sera were positive for HA, but not for HA1, of H1_2009_, which was much less than what was observed for dogs (Fig. 4 and 6). As a result, analysis of the Pearson correlation between the OD values obtained with the H1_2009_ HA- and HA1-ELISA (see Fig. S2) gave a higher value for dogs (*R*^2^ = 0.959) than for cats (*R*^2^ = 0.812). Furthermore, the dog and cat sera significantly differed (*P* = 0.0069) in the seroprevalence of antibodies specific for avian IAVs, which was higher in cats than in dogs.

## DISCUSSION

To date, several serological studies of IAV in cats and dogs have been performed, and seroprevalence of antibodies against several different IAV subtypes has been observed (reviewed in reference 14). Most of these studies are based on samples collected from animals in Asia and the United States. As a consequence, information regarding the circulation of IAV in cat and dog population in Europe is still limited. In the present study, we analyzed antibody responses in sera collected from different Dutch cat and dog cohorts against HA proteins of different IAV subtypes by applying a combination of newly developed serological assays that differ in their specificity and sensitivity. By using these different assays, we demonstrate a high seroprevalence of IAV-specific antibodies in sera from both species, particularly against the H1N1pdm09 virus. Several cats and dogs also displayed reactivity against avian IAVs, thereby indicating the potential of these animals to serve as an IAV mixing vessel.

IAV HA contains an immunodominant, but divergent, HA1 subunit and an immunosubdominant, but highly conserved HA2 subunit. The HA1 subunit is commonly used as antigen in a protein microarray format for assessing vaccination efficacy (42, 43). Recently, this method was also used for IAV seroprevalence studies in chicken or horse populations (44, 45). However, by only focusing on HA1, antibodies against more divergent strains might be missed. Therefore, in the present study, in addition to HA1, we also used complete HA ectodomains stabilized in their native trimeric conformation in ELISAs. The ELISA with trimeric HA allows detection of antibodies against epitopes present in both the HA1 and the HA2 subunit, which maximizes detection of IAV antibodies, including potential cross-reactive antibodies elicited by more distantly related HAs that fail to recognize HA1 but still target the conserved HA2 subunit. These ELISAs were complemented with highly specific HI assays, which specifically detect antibodies targeting epitopes surrounding the RBS. Combining complementary assays differing in specificity and sensitivity allowed us to observe a high seroprevalence of IAV-specific antibodies in cats and dogs, which for several animals could be confirmed by highly specific HI assays. While the assays developed within this study are not likely to provide help with clinical decision making, they will be of use for research and public health investigations. Of note, an often-used commercially available ELISA, in which the highly conserved nucleoprotein is used as antigen, suffered from very low sensitivity compared to our in-house HA and HA1 ELISAs, which partly may explain the low seroprevalence normally observed in studies using similar nucleoprotein-based ELISAs (15, 41).

By multivalently presenting HA on nanoparticles we developed a reliable HI assay. Traditionally, HI is performed with live or inactive virus particles, for which virus propagation is necessary. Obviously, this cannot be easily applied in all laboratories, particularly when biosafety level 3 containment is required. By using recombinant protein-based assays, this issue may be resolved, resulting in HI assay platforms that are easy to perform and standardize. Recently, a similar HA-NP design was used for immunization studies, and effective presentation of HA proteins was confirmed by electron microscopy (46). A comparable approach was also conducted for Middle East respiratory syndrome coronavirus (MERS-CoV) serology, where the sialic acid binding MERS-CoV spike S1^A^ domain was presented on NPs to perform an HI assay (47). In the present study, we showed that results obtained with HA-NPs and virus particles are nearly identical (see Table S3), indicating that the HI assay with HA-NPs may be used as an alternative for the HI assay with virus particles, which is currently regarded as the gold standard (48).

We analyzed the prevalence of IAV-specific antibodies in cat and dog samples from different cohorts. In the 2016 and 2019 cat cohorts, positive reactivity was noticed with HA of each of the seven IAV strains via HA ELISA, with most responses against H1_2009_ (Fig. 4 and 5). Similarly, the majority of seropositivity in the 2019 dog cohort was observed for H1_2009_. Both for cats and dogs, H1 reactivity was significantly higher (*P*_cat_ = 0.0141, *P*_dog_ = 0.0038) in samples collected after 2009 than in samples collected prior to 2009, whereas reactivities against other subtypes were not significantly higher (see Tables S5 and S6 in the supplemental material). The H1 reactivity in post-2009 cohorts is also significantly higher (*P* < 0.0001 in both cases) than the H3 reactivity (Table 1). This might be explained by H1N1pdm09 being more capable of crossing the host species barrier than the human H1N1 virus that circulated prior to 2009 or the human H3N2 virus. While the reason for this difference is not known, it may be related in part to differences in the receptor-binding properties of these different human viruses, with H1N1pdm09 being able to bind to α2,3-linked, in addition to α2,6-linked, sialic acid receptors, which contrasts with pre-2009 H1N1 that poorly binds α2,3-sialic acid (49).

We provide the first serological evidence of infection of cats with H3, H5, H7, and H9 subtypes in Europe. Such observations are in agreement with seroprevalence studies of cat sera conducted in Asia and the United States (41, 50, 51). Although a significantly lower (*P* = 0.0069) seroprevalence of antibodies against avian IAVs could be detected in dogs compared to cats (Table 1), in some dog sera antibody responses against H5 and H7 were detected using different assays, including HI for H7. This is the first evidence of infection with H5 and H7 subtype viruses in European dogs. The seropositivity of cat and dogs is likely due to spillover of avian IAVs from birds to cats and dogs. Apparently, this happens more often for cats than for dogs, in agreement with their predatory behavior. Interestingly, both the H5- and the H7-positive sera are from hunting dogs that might chase birds. The presence of H7-specific antibodies in dogs has so far only been reported once for one dog in Africa (52).

In conclusion, our findings demonstrate the value of using comprehensive serological assays to analyze antibodies against IAVs and for improved serosurveillance of IAV infections. Although we only analyzed cat and dog sera, it will be of interest to analyze to what extent these assays will be applicable for the serosurveillance of (zoonotic) IAV infections in humans, which is currently particularly being done using HA1-based assays (53). Our results indicate that infection of cats and dogs with several subtypes of IAVs is prevalent. This emphasizes the potential role of both animal species as mixing vessels for IAVs, with the possibility of the emergence of mutated/reassorted viruses with increased zoonotic potential. Recurrent epidemiological surveillance for influenza infections among cats and dogs is needed, which could serve as an early cautioning system for human and animal threats.

## Supplementary Material

Supplemental file 1
