# Supplementary material for: Serological Screening of Influenza A Virus Antibodies in Cats and Dogs Indicates Frequent Infection with Different Subtypes
Source: J Clin Microbiol. 2020 Oct 21;58(11):e01689-20. doi: 10.1128/JCM.01689-20 (PMC7587082; doi:10.1128/JCM.01689-20)
Supplement: Supplemental file 1 [file JCM.01689-20-s0001.pdf]

Supplementary materials

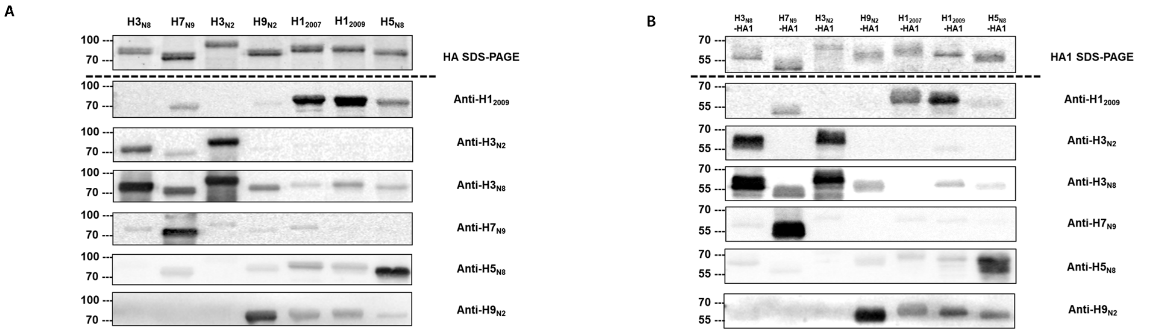

Figure S1. Reactivities of reference antisera against different HA (A) and HA1 (B) proteins analyzed by western blot analysis.

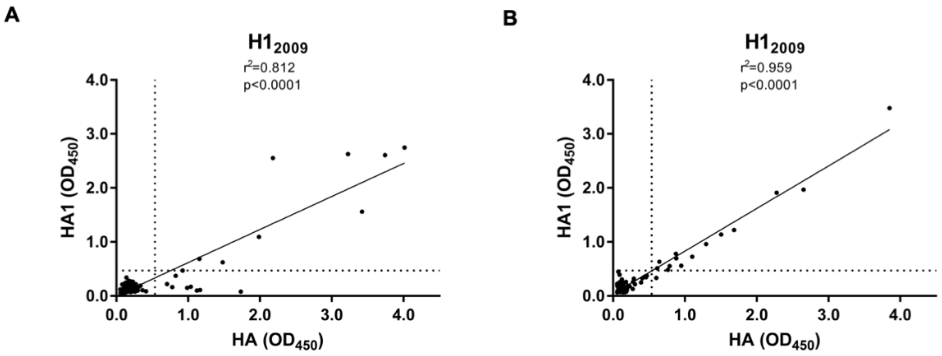

Figure S2. Correlation between the OD values obtained with HA and HA1 ELISA of $H1_{2009}$. (A) Cat serum samples from 2016 cohort; (B) dog serum samples from 2019 cohort. Correlation and regression coefficients were shown above the figures.

A

cat sample	HA: $H1_{2007}$	HA: $H1_{2009}$	HA: $H3_{N2}$	HA: $H3_{N8}$	HA: $H5_{N8}$	HA: $H7_{N9}$	HA: $H9_{N2}$	HA1: $H1_{2007}$-HA1	HA1: $H1_{2009}$-HA1	HA1: $H3_{N2}$-HA1	HA1: $H3_{N8}$-HA1	HA1: $H5_{N8}$-HA1	HA1: $H7_{N9}$-HA1	HA1: $H9_{N2}$-HA1	HA-NPs: $H1_{2007}$	HA-NPs: $H1_{2009}$	HA-NPs: $H3_{N2}$	HA-NPs: $H3_{N8}$	HA-NPs: $H5_{N8}$	HA-NPs: $H7_{N9}$	HA-NPs: $H9_{N2}$
kb125	4.39	4.79	0.30	1.75	3.45	0.24	0.19	0.43	0.55	0.63	0.55	0.47	0.45	0.29	0	0	0	0	0	0	0
c11729	2.02	3.32	0.22	0.21	1.07	0.46	0.14	0.60	1.26	0.39	0.39	0.34	0.31	0.17	0	0	0	0	0	0	0
c11703	0.26	0.24	0.36	1.05	1.45	0.23	0.19	0.61	0.49	0.68	0.56	1.92	0.54	0.30	0	0	0	0	0	0	0

OD450 value vs. positive cut-off: < 1; 1- 2; >2- 4; > 4

HI titer: < 20; 20; 40; 80; > 80

B

Dog sample	$H1_{2007}$	$H1_{2009}$	$H3_{N2}$	$H3_{N8}$	$H5_{N8}$	$H7_{N9}$	$H9_{N2}$	$H1_{2007}$-HA1	$H1_{2009}$-HA1	$H3_{N2}$-HA1	$H3_{N8}$-HA1	$H5_{N8}$-HA1	$H7_{N9}$-HA1	$H9_{N2}$-HA1	$H1_{2007}$	$H1_{2009}$	$H3_{N2}$	$H3_{N8}$	$H5_{N8}$	$H7_{N9}$	$H9_{N2}$
52	0.23	0.20	0.19	0.28	0.18	3.58	0.16	0.19	0.24	0.22	0.24	0.18	4.41	0.17	0	0	0	0	0	40	0
96	0.37	0.37	0.32	0.39	0.35	2.82	0.39	0.15	0.18	0.15	0.17	0.14	1.91	0.19	0	0	0	0	0	80	0

OD450 value vs. positive cut-off: < 1; 1- 2; >2- 4; > 4

HI titer: < 20; 20; 40; 80; > 80

Figure S3. Reactivity of cat (A) and dog (B) serum samples collected before 2009 against different HAs as measured by HA ELISA, HA1 ELISA and nanoparticle-based HI. ELISA results are expressed as ratios of OD450 value divided by the positive cut-off (OD450 value vs positive cut-off).

Table S1. Half maximal effective concentration (EC_{50}) determined of each reference serum against corresponding HA and HA1 proteins. Results are expressed as dilution ratios.

	anti-$H1_{2009}$	anti-$H3_{N2}$	anti-$H3_{N8}$	anti-$H5_{N8}$	anti-$H7_{N9}$	anti-$H9_{N2}$
HA	1:1416	1:232	1:2847	1:2710	1:14371	1:182
HA1	1:838	1:41	1:2547	1:2632	1:15404	1:70

Table S2. Numbers of all HA positive (n=29) and 31 negative cat samples from 2016 cohort with different combinations of reactivity observed. Positive reactions are colored in orange.

	HA-ELISA							HA1-ELISA							HA-NPs							ID Screen®	Number of cats (Total=29)
Row nr.	$H1_{2007}$	$H1_{2009}$	$H3_{N2}$	$H3_{N8}$	$H5_{N8}$	$H7_{N9}$	$H9_{N2}$	$H1_{2007}$	$H1_{2009}$	$H3_{N2}$	$H3_{N8}$	$H5_{N8}$	$H7_{N9}$	$H9_{N2}$	$H1_{2007}$	$H1_{2009}$	$H3_{N2}$	$H3_{N8}$	$H5_{N8}$	$H7_{N9}$	$H9_{N2}$		
**A**		+							+							+						+	3
**B**		+							+							+							2
**C**		+		+					+		+					+							1
**D**	+	+			+				+							+							1
**E**	+	+							+														1
**F**		+							+														1
**G**	+	+		+	+																		1
**H**	+	+			+																		1
**I**	+	+				+																	2
**J**		+																					4
**K**	+							+															1
**L**			+																				1
**M**			+		+							+											1
**N**				+				+			+	+						+					1
**O**					+							+							+				1
**P**					+							+											1
**Q**					+														+				1
**R**					+																		2
**S**						+																	2
**T**							+																1
**U**																							31

Table S3. Comparison of hemagglutination inhibition (HI) titers of cat serum samples against $H1_{2009}$-NPs and H1N1 virus particles. Negative sera (HI titer <20), are represented as 0 in the table.

Characteristic	number	$H1_{2009}$-NPs	H1N1
Shelter cat serum samples (2016 cohort)	UFB 33	80	80
	UFB 93	80	80
	UFB 22	20	20
	UFB 74	40	40
	UFB 277	40	20
	UFB 279	20	20
	UFB 31	40	40
	UFB 11	0	0
	UFB 17	0	0
	UFB 18	0	0
	UFB 14	0	0
	UFB 23	0	0
FCoV specific sera derived from SPF cats	cat 14	0	0
	cat 15	0	0
	cat 91	0	0
	cat 93	0	0
	cat 95	0	0
	cat 115	0	0
	cat 131	0	0

Abbreviations: H1N1, Influenza virus A/Netherlands/602/2009; $H1_{2009}$-NPs, SpyCatcher-mi3: $H1_{2009}$-SpyTag nanoparticles; FCoV, feline coronavirus; SPF, specific pathogen free

Table S4. Numbers of all HA positive (n=21) and 14 negative dog samples from 2019 cohort and different combinations of reactivity observed. Positive reactions are colored in orange.

Row nr.	HA-ELISA							HA1-ELISA							HA-NPs							ID Screen®	Number of dogs (Total=21)
	$H1_{2007}$	$H1_{2009}$	$H3_{N2}$	$H3_{N8}$	$H5_{N8}$	$H7_{N9}$	$H9_{N2}$	$H1_{2007}$	$H1_{2009}$	$H3_{N2}$	$H3_{N8}$	$H5_{N8}$	$H7_{N9}$	$H9_{N2}$	$H1_{2007}$	$H1_{2009}$	$H3_{N2}$	$H3_{N8}$	$H5_{N8}$	$H7_{N9}$	$H9_{N2}$		
**A**		■			■				■							■						■	1
**B**		■							■			■				■							1
**C**		■							■							■						■	2
**D**		■							■							■							7
**E**		■							■														2
**F**		■							■													■	1
**G**		■														■							2
**H**		■																					3
**I**					■							■											1
**J**						■							■							■			1
**K**																							14

Table S5. Summary of antibody responses against each HA by HA ELISA in pre-2009 or post-2009 cat cohorts.

	Pre-2009				Post-2009				Pre-2009 vs. Post-2009
**HA**	**Sample number**	**Positive number**	**Prevalence[a]**	**95% CI[b]**	**Sample number**	**Positive number**	**Prevalence[a]**	**95% CI[b]**	***p* value[c]**
$H1_{2007}$	68	2	0.029	0.002-0.107	253	12	0.047	0.027-0.082	0.5275
**$H1_{2009}$**	**68**	**2**	**0.029**	**0.002-0.107**	**253**	**37**	**0.146**	**0.108-0.195**	**0.0141**
$H3_{N2}$	68	0	0	0.000-0.064	253	5	0.020	0.007-0.047	0.2464
$H3_{N8}$	68	2	0.029	0.002-0.107	253	4	0.016	0.005-0.041	0.4664
$H5_{N8}$	68	3	0.044	0.010-0.127	253	11	0.043	0.024-0.077	0.9823
$H7_{N9}$	68	0	0	0.000-0.064	253	6	0.024	0.010-0.052	0.2041
$H9_{N2}$	68	0	0	0.000-0.064	253	6	0.024	0.010-0.052	0.2041

[a]prevalence =positive number/sample number

[b]95%CI: 95% confidence interval (computed by the modified Wald method)

[c]*p* value: represent the significance of the difference of the prevalence between two cohorts (Chi-square test)

Table S6. Summary of antibody responses against each HA by HA ELISA in pre-2009 or post-2009 dog cohorts.

	Pre-2009				Post-2009				Pre-2009 vs. Post-2009
**HA**	**Sample number**	**Positive number**	**Prevalence[a]**	**95% CI[b]**	**Sample number**	**Positive number**	**Prevalence[a]**	**95% CI[b]**	***p* value[c]**
$H1_{2007}$	68	0	0	0.000-0.064	154	0	0	0.000-0.029	n/a
**$H1_{2009}$**	**68**	**0**	**0**	**0.000-0.064**	**154**	**19**	**0.123**	**0.080-0.185**	**0.0038**
$H3_{N2}$	68	0	0	0.000-0.064	154	0	0	0.000-0.029	n/a
$H3_{N8}$	68	0	0	0.000-0.064	154	0	0	0.000-0.029	n/a
$H5_{N8}$	68	0	0	0.000-0.064	154	2	0.013	0.001-0.049	0.3471
$H7_{N9}$	68	2	0.029	0.002-0.107	154	1	0.007	<0.0001-0.040	0.1758
$H9_{N2}$	68	0	0	0.000-0.064	154	0	0	0.000-0.029	n/a

[a]prevalence =positive number/sample number
[b]95%CI: 95% confidence interval (computed by the modified Wald method)
[c]*p* value: represent the significance of the difference of the prevalence between two cohorts (Chi-square test)
